# On the Disease of the Eye, Called Amaurosis, or Gutta Serena; Extracted from an Essay of Professor Arnemann, of Goettingen

**Published:** 1799-03

**Authors:** 


### *On the Difeafe of the Eye, called Amaurofis, or Gutta Serena; extracted from an Effay of Profeffor* ARNEMANN, *of Goettingen.*

IT is with great fatisfaction we obferve the progreffive improvement of the medical art, and are led to admire and applaud human ingenuity, when difeafes themfelves are now-a-days employed to reduce the catalogue of human mifery. We inoculate the fmall-pox, in order to deprive this dangerous enemy to the human race of its deftructive power; we purpofely reproduce the itch, *plica polonica*, and fimilar diforders, with a view to obviate more fatal maladies, and—an ARNEMANN even excites vertigo in his patients, the more effectually to extirpate this fpecies of blindnefs. And yet, confidering the fubject in an extenfive point of view, it muft be confeffed, that phyficians have ever adopted it as a ftanding maxim, to remove or fupprefs the more important difeafes, by artificially inducing others of lefs dangerous tendency. What elfe can we call diarrhœas, the effects of diaphoretics, and even thofe of emetics, but flight diftempers created or produced for the fake of obviating greater ones? Notwithftanding this, Arnemann is, within our knowledge, unqueftionably the firft who conceived the original idea of ftrengthening and reftoring its tone to the eye, by exercifing it with vertiginous or rotatory commotions of the head*.

This

* This effect may be produced by placing the patient on a whirling table, with his head at various diftances from the centre. B.

This ingenious propofal is certainly intitled to refpectable attention; neither is it improbable, that the fame or a fimilar method might be introduced with advantage, in nervous and other affections of the head, in which the modern imitators in phyfic find themfelves frequently baffled.

The determination of the blood towards the head, which accompanies giddinefs, affords fome rational grounds for this conjecture, and may well juftify the attempt. An increafed accefs of the fluids to the relaxed parts muft affuredly be productive of beneficial effects, and particularly in cafes where this relaxation originates in torpor. A remedy of this nature will then act properly as an exciting mean; inafmuch as the accefs of the fluids roufes the vital power. Here then we fhould enquire, whether the intervention of that circumftance (viz. the accefs of the fluids) offers any particular advantages in the ufe of this remedy?—As vertigo is, in every inftance, a morbid indication, in the language of phyficians, it may well be confidered a decifive fymptom, that fuch a remedy operates on the weak nerves, and inundates them with fluids in a preternatural degree; but we are not thence entitled to prefer the application of one *excitatory*, to another which is not of the vertiginous kind; as for example, the ufe of foporific remedies. Dr. Arnemann, therefore, has very properly remarked, that the excitation of vertigo, if it prove to be of falutary effect, is a favourable fign; but it is not, on that account, to be confidered as a co-operating caufe, in removing the complaint.

This diftinction is the more neceffary to be attended to, as the choice and application of the remedy are thereby determined. With all the aid of theory, we can find no fatisfactory arguments to recommend the vertiginous remedies, as fuch, in the cure of the amaurofis. Their fudden, and as it were momentary, effect, indicates either a greater degree of debility, or an increafed circulation of the blood; and although it may be judged a favourable fymptom in the latter cafe, yet it is not even then by any means a material circumftance. The circulation of the blood is by thefe remedies only fo far promoted, as they are in their nature ftimulating, and not from the effect produced by them, viz. vertigo. The cafe would be different, if giddinefs *of itfelf* were neceffarily accompanied with increafed commotions in the whole fyftem; but this is obferved to take place only in vertigo from plethora. The greater number of fpecies of vertigo are, on the contrary, attended with a weak, unequal, hyfteric pulfe, and generally with palenefs of the face, and a fenfation of cold.

Thefe objections, to which the now propofed method of cure in amaurofis, is liable in theory, are by no means intended to depreciate the juft praife to which the author has an undoubted claim, for the *practical* difcovery of another remedy, againft the fame diforder. It briefly confifts of *camphor*, with

*with, and without, belladonna:* an excellent tonic and ſtrengthening medicine to the eyes, and which has frequently done eſſential ſervice, even in the dangerous ſpecies of cataract. But let it be remembered here, that camphor and belladonna can be productive of good only in caſes of real debility, ariſing from want of excitation, or in *aſthenia*; while, on the contrary, in plethoric weakneſs and paralyſis, they muſt neceſſarily be detrimental to the patient, and aggravate rather than diminiſh the ſymptoms of the diſeaſe.

The ſalutary effect of camphor, adminiſtered with and without belladonna, has been ſtrongly confirmed in the practice of the learned Profeſſor, who appears to have preſcribed it ſucceſsfully, in a variety of caſes. The former was directed to be taken in doſes, from two to ſix grains, generally twice a day; the belladonna from one to two grains at a time, two doſes every day. —In one particular inſtance of amauroſis, as deſcribed by the Profeſſor, and which originated from external injury, other practitioners would, no doubt, have preferred the flowers of the *arnica**, to either camphor or belladonna; as

* The *Arnica montana* of Linnæus, or German leopard's bane, is one of the most deleterious vegetables we are acquainted with. It has long been known and used by the common people in Germany, as an efficacious remedy for bruises and other external injuries, especially with a view to resolve and discuss extravasated and coagulated blood: hence, the Germans have given it the name of *Fallkraut*, or the herb for falls and bruises: Fehr was the first who published an account of the virtues of this plant, in the 9th and 10th vols. of the "*Ephemerides of the Imperial Society of Naturalists, at St. Petersburg*;" in which he calls it '*Panacea lapsorum*.' But on account of the violent stimulus which this remedy excites, so that the external use of it is generally attended with great anxiety, and frequently also with vomiting, it requires much precaution in the mode of exhibiting it. In most cases it is necessry, previous to its use, to take a few ounces of blood from the patient, to open the body by means of glysters and cooling laxatives, and if the inflammation should be considerable, to postpone the administration of the medicine, till the inflammatory diathesis be suppressed, or greatly mitigated.

The herb and flowers of the arnica are, to irritable habits, most conveniently given, in simple infusion. One drachm of the dried flowers, or double that quantity of the leaves, is infused in one quart of cold water, and a tea-cup full of this infusion is directed to be taken every two, three, or four hours through the day; this small dose ought to be gradually increased, according to circumstances, so that from one to *three*, or *four* drachms may be infused in *one* quart of water. On account of its volatile particles, this plant should never be *boiled*; and, for the same reason, the infusion ought to be made in *close vessels*. According to the opinion of the celebrated Dr. Crell, a cold infusion of the arnica is preferable to the strongest decoction; as the water of the former, in twenty-four hours, becomes as highly coloured and penetrating as the root itself.

Perhaps the want of success, together with the different effects of this vegetable, as observed by different practitioners, is chiefly to be ascribed to their inattention, with respect to the mode of its preparation, and the manner of its exhibition.—An useful as thefe flowers, by the concurrent teftimony of practitioners, have acquired the reputation almoft of a fpecific, and as they have lately been employed with fingular fuccefs by Dr. COLLIN of Vienna, in patients afflicted with the cataract.—We cannot, however, omit to obferve in this place, that other medical practitioners, particularly Dr. RICHTER of Goettingen, and Dr. GESENIUS of Nordhaufen, in Germany, have lately been much difappointed in the fpecific virtues thefe flowers are faid to poffefs, either in cafes of general paralyfis, or gutta ferena.

useful addition to the above directed infusion, are the leaves and flowers of the milfoil, *Achillea Millefolium*, Lin. which eminently possess the property of diminishiug the violent, stimulating effects, generally following the use of the arnica. W.

